# Neuroprogression as an Illness Trajectory in Bipolar Disorder: A Selective Review of the Current Literature

**DOI:** 10.3390/brainsci11020276

**Published:** 2021-02-23

**Authors:** Gianluca Serafini, Matteo Pardini, Fiammetta Monacelli, Beatrice Orso, Nicola Girtler, Andrea Brugnolo, Mario Amore, Flavio Nobili, Disease Management Team on Dementia of the IRCCS Ospedale Policlinico San Martino

**Affiliations:** 1 Department of Neuroscience, Rehabilitation, Ophthalmology, Genetics, Maternal and Child Health DINOGMI, Section of Psychiatry, University of Genoa, 16132 Genoa, Italy; Andrea.Brugnolo@unige.it (A.B.); mario.amore@unige.it (M.A.); 2 IRCCS Ospedale Policlinico San Martino, 16132 Genoa, Italy; matteo.pardini@gmail.com (M.P.); fiammetta.monacelli@unige.it (F.M.); nicolagirtler@unige.it (N.G.); flavio.nobili@unige.it (F.N.); 3 Department of Neuroscience, Rehabilitation, Ophthalmology, Genetics, Maternal and Child Health DINOGMI, Section of Neurology, University of Genoa, 16132 Genoa, Italy; beatrice27orso@gmail.com; 4 Department of Internal Medicine and Medical Specialties, DIMI, Section of Geriatrics, 16132 Genoa, Italy

**Keywords:** bipolar disorder, illness trajectory, neuroprogression, illness course, illness outcome

## Abstract

Bipolar disorder (BD) is a chronic and disabling psychiatric condition that is linked to significant disability and psychosocial impairment. Although current neuropsychological, molecular, and neuroimaging evidence support the existence of neuroprogression and its effects on the course and outcome of this condition, whether and to what extent neuroprogressive changes may impact the illness trajectory is still poorly understood. Thus, this selective review was aimed toward comprehensively and critically investigating the link between BD and neurodegeneration based on the currently available evidence. According to the most relevant findings of the present review, most of the existing neuropsychological, neuroimaging, and molecular evidence demonstrates the existence of neuroprogression, at least in a subgroup of BD patients. These studies mainly focused on the most relevant effects of neuroprogression on the course and outcome of BD. The main implications of this assumption are discussed in light of specific shortcomings/limitations, such as the inability to carry out a meta-analysis, the inclusion of studies with small sample sizes, retrospective study designs, and different longitudinal investigations at various time points.

## 1. Introduction

Bipolar disorder (BD) is a chronic and disabling psychiatric condition with a lifetime prevalence of more than 2% [1]. This clinically severe disorder currently represents one of the leading causes of disability worldwide, with a very important impact on individuals, families, and society [2]. While episodes of mania in BD may be appropriately managed in the clinical practice, recurrent depressive episodes currently represent a major challenge for clinicians and are usually associated with chronicity and illness neuroprogression [3]. The term “neuroprogression” is used here to explain changes within brain structures (i.e., atrophy and volume loss, as well as cognitive decline and vulnerability to psychological stress) and their association with a more severe course of illness [4,5]. In fact, neuroprogression seems to enhance the vulnerability to neurodegenerative processes since it involves changes in grey matter volume, together with Lewy-related alpha-synucleinopathy [6]. Importantly, both syndromal or subsyndromal depressive symptoms may contribute to the significant morbidity and mortality reported in BD and may account for a growing functional impairment over time [7].

Progressive changes in brain structure and cellular function, mainly supporting the general assumption of neuroprogression, have been described in studies including patients with recurrent episodes of affective disorders [8]. In particular, a longer illness duration in medicated BD patients has been linked to an abnormally reduced cortical thickness of specific brain regions, such as the prefrontal cortex playing a fundamental role in stress regulation [9]. 

Overall, the neuroprogression hypothesis supposes that the recurrence of mood episodes might promote cumulative damage in neural cells (e.g., neurons and glia), affecting the integrity of neural circuits and enhancing brain vulnerability to developing future illness episodes [10]. Multiple dysfunctions affecting both cellular resilience and neuroplasticity have been specifically evoked in this regard [8,11,12]. Berk et al. [8] proposed that the existence of epigenetic changes, mitochondrial dysfunctions, abnormally impaired neural pathways related to neuroplasticity, neuroinflammation, and increased oxidative and nitrosative stress levels in subjects with BD further supported the notion of neuroprogression over time. In particular, the enhanced vulnerability to developing neurodegenerative processes seems to be associated with neuroinflammation, higher pro-inflammatory cytokine levels, increased oxidative stress, mitochondrial dysfunctions, and abnormally reduced neurotrophic factors [13,14,15,16,17,18,19]. Clinically, the higher and recurrent nature of mood episodes and hospitalizations, the enhanced suicide risk, and poor response to commonly available treatments over the illness course that are directly related to chronicity confirm the neuroprogressive hypothesis underlying this condition [20,21]. 

Some researchers have hypothesized that BD may develop a progressive path over time in particular in a subgroup of BD patients who showed initial subtle cognitive impairments and were more likely to develop chronicity [22,23]. In addition, several neuroimaging studies showed specific brain abnormalities in BD in order to validate the neuroprogressive hypothesis underlying this condition, with the most relevant findings referring to the presence of both regional structural and functional alterations, predominantly in the striatal–insular–thalamic and temporo–parietal associated regions [24], at least in a specific subgroup of subjects. 

However, the course and illness trajectories of BD may be quite heterogeneous, as demonstrated by the existence of a subgroup of patients who usually preserve cognitive and psychosocial functioning and productivity throughout the illness. Thus, the present review aimed to comprehensively and critically investigate the current neuropsychological, neuroimaging, and molecular evidence about the link between BD and neurodegeneration and provide an updated overview of the available evidence about this topic. In particular, this review focused mainly on the impact of neuroprogression on both the course and illness trajectory of BD. 

## 2. Methods

A comprehensive search strategy was used in order to detect the most relevant available studies. To provide a critical review concerning the link between BD and neurodegeneration, we carried out searches in PubMed/Medline, Scopus, and ScienceDirect to identify relevant papers during the period between 1990 and November 2020. The following terms were used in the present review: ‘‘BD’’ OR ‘‘Bipolar Disorder” AND “cognitive dysfunctions” OR “cognitive impairment” AND “neuroimaging abnormalities” AND “neurodegeneration” OR “neuroprogression” AND “lithium”. A first literature search was performed; only articles in peer-reviewed journals and written in the English language were included. In addition, the reference lists of the full-text articles were manually checked in order to identify other studies and publications that were cross-referenced to find further existing articles on the same topic. 

## 3. Results

### 3.1. Neurocognitive Correlates and Illness Neuroprogression 

Cognitive dysfunctions represent a relevant part of the clinical conceptualization of BD, with many studies to date that have demonstrated the presence of cognitive dysfunctions in BD during euthymia. Cognitive domains that have been examined in bipolar patients include executive function, memory, attention, psychomotor speed, and social cognition [25]. Martínez-Arán et al. [23] performed a cross-sectional study comparing bipolar patients and healthy controls (HC). After controlling for the effect of subsyndromal depressive symptoms and premorbid IQ, the bipolar group showed, when compared to controls, significant impairments in verbal memory and executive functions. On the other hand, Clark et al. [25], Deckersbach and colleagues [26], and Martínez-Arán et al. [23] found that a poorer performance in delayed verbal memory correlated with the number of manic episodes in treated BD patients, suggesting that the experience of manic episodes appeared to be associated with poorer retention. 

Another cross-sectional study by Robinson et al. [27] showed an association between a worse prior course of illness and neuropsychological dysfunctions, in particular, with long-delay verbal memory. Conversely, the decline in executive functioning seems related to a worse illness progression. Torres et al. [28] reported that patients with multiple mood episodes were more impaired, both in verbal memory and executive functions, than those who recovered from their first manic episode. 

According to these findings, each recurrence has a cost in terms of cognitive impairment and, therefore, recurrences may be interpreted as evidence of neuroprogression in BD [8,29,30,31,32,33]. Based on this model, cognitive deficits were considered as hallmarks of the mentioned model of “neuroprogression” that support the relationship between the worsening of cognitive performance and the number of critical depressive and manic episodes [34]. In fact, the progressive decrement in cognitive functioning of those patients could be explained by alterations in neurotrophins, inflammation mediators, and oxidative stress occurring as a consequence of affective episodes [35]. Concomitantly, the neuroprogression hypothesis was included as the cornerstone of staging models of BD that have been proposed in recent years [16,34,36].

The number of prior illness episodes was found to be associated with cognitive impairment [27], quality of life, disability, and the severity of symptoms [37]. However, subjects with BD type I who were euthymic for one year after the illness onset had better cognitive performances in comparison with subjects with recurrent mood alterations [38]. Furthermore, the history of BD significantly increased the risk of dementia in older adults, with an odds ratio of 2.36 [39]. These data were confirmed by a recent meta-analysis that found a direct relationship between the number of mood episodes and the risk of developing dementia [40]. 

Despite the existence of the aforementioned evidence, there are conflicting assumptions on the nature of cognitive impairments and their relationship with illness progression. El-Badri et al. [41] found a negative correlation between the total number of episodes and executive dysfunctions, such as the performance on an oral word list generation task and Tower of London task, while Kieseppä et al. [42] did not find a significant relationship between the number of episodes and any neuropsychological measures. Longitudinal studies demonstrated that the relation between the clinical course of illness and cognitive impairments was inconclusive; thus, cognitive impairment may be considered the cause rather than the consequence of the clinical course [43]. According to Sparding et al. [44], only 30% of those with clinically stable BD demonstrated cognitive abilities that significantly differ from HC, whereas the other groups showed a cognitive performance similar to HC. According to Gildengers and colleagues [45], among patients over fifty years, the BD group did not show an accelerated decline compared to controls, even if at baseline, their cognitive performance was lower than that of HC. Furthermore, an extensive revision and meta-analysis of longitudinal studies did not sustain the progressive nature of cognitive deficits in BD [46]. 

Although prominent cognitive deficits have been documented in BD, the prevalence of cognitive dysfunctions in BD studies is variable. Specifically, the alterations of executive functions range from 5.3 to 57.7%, attention/working memory deficits range from 9.6 to 51.9%, speed/reactions impairment ranges from 23.3 to 44.2%, verbal memory reduction ranges from 8.2 to 42.1%, and visual memory alteration ranges from 11.5 to 32.9%. Another source of variability concerns the assessment of neurocognitive functions, which is heterogeneous among the selected studies due to the different cognitive areas that were selected, and the tests were adopted with their specific cut-offs [47]. 

Since there is a remarkable heterogeneity regarding the cognitive outcomes, existing evidence suggests caution when interpreting data and the need for more accurate characterization of different phenotypes associated with BD. In particular, different factors may be related to this variability, and their identification might help to better define different phenotypes and reduce this heterogeneity [48]. There are several promising studies that may pave the way to a better definition of the different endophenotypes of BD. Impairments in social cognition are of relatively new interest but are still important for better understanding the social functioning of these patients. In a recent systematic review [49], the role of impairments in the theory of mind, emotion perception, and emotion processing have been highlighted. These authors showed that BD patients displayed significantly impaired recognition, identification, and discrimination of facial emotions compared with HC [50,51]. Lahera and colleagues [52] reported that depressive symptoms in BD may modulate emotion processing and functional outcomes. Another important factor that has been evaluated is the prevalent polarity of mood disorders (manic vs. depressive) that may play a relevant role in cognitive functioning. In fact, according to Belizario et al. [53], predominant manic polarity in BD is associated, regardless of psychotic symptoms, with a greater cognitive impairment compared to BD with prevalent depressive polarity. 

The analysis of a large dataset using machine learning techniques represents another promising direction for research on BD since it can integrate different clinical and functional data, cognitive abilities, and multiple levels of biological data. According to Librenza-Garcia et al. [54], machine learning may overcome the heterogeneity of BD samples and identify which data structures are useful for providing insights into differential diagnosis, customized treatment, and prognosis orientation. The studies collected here converge on the use of biomarkers, especially structural and functional neuroimaging, for the characterization of BD, as well as other psychiatric disorders and HC [54]. The combination of neurocognitive instruments and neuroimaging techniques, using both supervised and unsupervised machine learning techniques, may distinguish biologically meaningful clinical phenotypes of BD with a very high level of accuracy [55]. Table 1 summarized the most relevant neurocognitive correlates of neurodegeneration in BD.

### 3.2. Neuroimaging Evidence and Neuroprogression in BD: An Exciting Journey through the History of This Link

Neuroimaging evidence was proposed as a tool to validate the neuroprogressive hypothesis of BD [56,57]. In the past thirty years, several global and regional metrics of grey matter volume losses have been explored as possible biomarkers of neuroprogression in BD.

The majority of older studies focused on whole-brain metrics, which, compared to regional measures, are arguably more robust and easier to compute, even if they are not able to detect focal structural alterations. The study of Rieder and colleagues [58] provided some of the first evidence of neuroprogression in BD, showing a significant association between normalized ventricular volume (a widely used metric of brain volume loss) as assessed using computerized tomography (CT) and age, albeit only in subjects of 50 years of age or older, irrespective of their clinical diagnosis (these data were observed even in subjects with schizophrenia and schizoaffective disorder). While from a current perspective, this study presents a number of limitations, including the coarseness of the used neuroimaging metric, it can rightly be considered highly significant, after taking into account its temporal context. Various cross-sectional studies have been conducted in BD concerning the association between whole-brain volume metrics and age, as well as other proxy metrics of disease exposure, such as disease duration and the number of untreated affective episodes, with conflicting evidence mainly being due to small sample sizes and differences in recruitment strategies [59]. However, given the aforementioned robustness of whole-brain atrophy measures, the development of international consortia that were aimed at pooling hundreds of volumetric scans in collaborative analyses tried to overcome these caveats. Using this strategy, Hallahan and colleagues [60] pooled magnetic resonance imaging (MRI) data from 321 patients with BD type I and 442 HC; they showed a robust association between disease duration and whole-brain volume loss, independently from age of disease onset. While the large sample size represents a significant strength of the study, this finding needs to be interpreted with caution, especially when considering the cross-sectional study design. Unfortunately, longitudinal studies of whole-brain volume have to date failed to reliably demonstrate an association between BD and the acceleration of atrophy [61]; this may be due to the fact that an observation limited to few years in relatively young subjects is presumably inadequate to capture subtle changes in a coarse metric such as whole-brain volume. In association with whole-brain measures, regional metrics may also be computed using volumetric MRI. Compared to whole-brain measures, the quantification of regional volumes is less straightforward, for instance, regarding the choice of the parcellation approach used to identify specific cortical regions or the impact of voxel size on volume assessment accuracy, but they are arguably more informative regarding the underlying pathological changes [62].

Overall, regional studies stressed the presence of regional structural abnormalities in BD, especially in fronto–temporo–striatal regions. Regarding the prefrontal cortex, a number of longitudinal studies pointed to its involvement in BD. Progressive prefrontal cortex volume loss has been described in BD adolescents over a relatively short time frame (i.e., two years) [63], mainly in association with disease severity as assessed with the frequency of hospitalizations [64]. While these studies reported a decrease in regional grey matter volume, several reports showed an increase in grey matter volume over time, at least in some facets of the prefrontal cortex (e.g., ventrolateral prefrontal cortex of young BD patients in two longitudinal independent studies [65,66]). Given the pathological evidence of reduced prefrontal neural density in BD subjects in at least some post-mortem studies, these findings suggested that the trajectory of BD-associated prefrontal volume changes was not linear over time, but probably U-shaped, thus prompting caution in the interpretation of negative findings in cross-sectional studies. The observations for temporal lobe structures, albeit sketchier, are in line with the prefrontal cortex findings, pointing to a relative increase in grey matter compared to controls [59,60]. Non-linear changes in grey matter volumes have also been described in subcortical structures. Indeed, early cross-sectional studies presented evidence of amygdala and striatum enlargement at BD onset [67], with other studies pointing to the relative stability of the amygdala volume over time in adults with BD [68]. Progressive changes in grey matter volumes have been observed in other subcortical structures of BD patients, such as the hippocampus [68], even if the interpretation of correlational findings in this structure is complicated by evidence that changes in hippocampal volumes during adolescence are sexually dimorphic, both in BD and HC [59].

The possibility of extracting regional volume metrics from structural MRI scans prompted a flurry of research on the association between cognition and local grey matter volumes in BD [69]. Overall, poor clinical outcomes were associated with more marked reductions in fronto–temporo–limbic network regions [70], with associations of temporal abnormalities with cognitive outcomes [68] and orbito–frontal abnormalities with impulsivity and higher suicidal behavior [71], even if these findings were not consistent across all published studies [72]. Despite the aforementioned heterogeneity in the reported results, which was at least partly due to differences in the study population characteristics, these findings point to the presence of regional structural alterations that are mainly located in the fronto–temporo–striatal regions, at least in a group of subjects with BD. Table 2 summarized the most relevant neuroimaging concerning neuroprogression in BD.

### 3.3. BD and Neurodegeneration: The Role of Lithium

**Figure 1:**
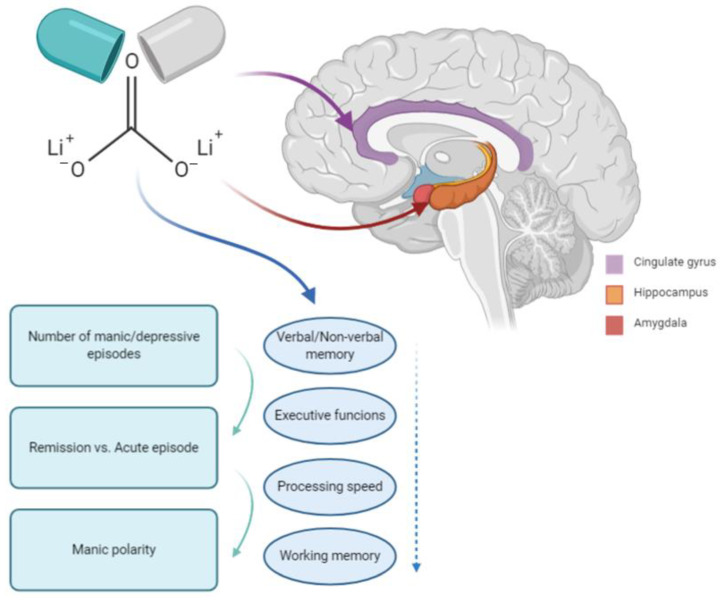
The role of lithium in bipolar disorder and neurodegeneration.

A key point in the investigation of the role of neurodegeneration in BD is represented by the impact of the use of chronic medications on brain volume. Indeed, both in vitro and neuroimaging studies point to a possible neuroprotective role for specific drugs that are commonly used to treat BD, such as lithium (Figure 1), both in affective disorders and Alzheimer’s disease [73]. While the putative mechanisms of the neuroprotective effect of lithium are only partly characterized, lithium has been shown to be able to increase levels of the neuroprotective protein B-cell lymphoma/leukemia-2 gene (*bcl-2*), promote neurite outgrowth [74], and mediate downstream effects of several neurotrophic factors [75].

Furthermore, according to Muneer [76], the enzyme glycogen synthase kinase 3 (GSK3) and its key upstream modulator, *Wnt*, have been proposed to be dysregulated in BD. *Wnt* molecules are able to regulate cell fate specification, proliferation, and neuronal morphology. Lithium, a well-known downregulator of GSK3β, which is the main inhibitor of the WNT/β-catenin pathway, was proposed to reverse the overactivity of GSK3 in BD, which is linked to a proinflammatory status, abnormally enhanced oxidative stress, the reduction of neurotrophic factors, and enhanced apoptosis [76,77]. Thus, the stimulation of the WNT/β-catenin after lithium treatment is presumed to be linked with the control of oxidative stress, neuroinflammation, and glutamatergic pathway.

Moreover, chronic lithium treatment is thought to dampen the impact of glutamate excitotoxicity [78]. In adult rodents, lithium produced a significant 25% increase in new cells in the dentate gyrus, demonstrating that this agent enhanced hippocampal neurogenesis [74]. Moreover, lithium has been shown to modulate the phosphorylation of tau, which plays a key role in a number of neurodegenerative diseases [73].

In addition, increasing myelin vulnerability resulting from the human brain’s abnormally persistent myelination was hypothesized in various neurodevelopmental conditions, such as schizophrenia and BD [79]. Specifically, prominent subcortical myelin damage and the association between bipolar susceptibility genes and WM volume and integrity deficits have been reported in BD [80,81]. Mood stabilizers, such as lithium, which are able to act as neurotransmitter-independent GSK3 inhibitors, may significantly promote the myelin repair/remyelination of subcortical white matter.

From a neuroimaging point of view, lithium use has been associated with preserved (and increased) regional volumes, both in both single- and multicenter studies. Indeed, compared with untreated subjects, BD patients treated with lithium presented with increased grey matter volume in vivo using structural MRI-based whole-brain measures and regional metrics [82,83] and the anterior cingulate [82]. Interestingly, the use of lithium was associated with an increase of a putative marker of neuronal integrity (N-acetyl-aspartate), which was found using MRI spectroscopy in both HC and BD patients, providing in vivo evidence of its possible neurotropic effect [84]. In the aforementioned collaborative study by Hallahan and colleagues [60], lithium use was associated with larger mean total, left, and right hippocampal volumes and total, left, and right amygdala volumes than both those patients not treated with lithium and HC. Moreover, in the same analysis, bipolar subjects not taking lithium presented with a smaller whole-brain volume compared with both the HC and bipolar subjects taking lithium. Interestingly, studies on the effects of lithium on regional grey matter metrics have shown a clinically relevant impact. Yucel and colleagues, for example [85], found a progressively increased volume of the hippocampus after treatment with lithium, in association with improved verbal memory. These findings suggested that treatment with lithium, at least moderately, may attenuate the loss of hippocampal grey matter.

The aforementioned in vitro and neuroimaging findings of a possible neuroprotective effect of lithium prompted several clinical studies aimed at assessing the impact of chronic lithium therapy on cognition in BD. Indeed, from a clinical perspective, lithium use is not without side effects, given the risk of renal problems and its ability to accumulate in tissues, such as the brain, possibly leading to neurological problems, including lithium-associated encephalopathies [86]. These concerns have led to an unjustified reduction of lithium use in clinical practice in recent years, which at least partially contrasted with the mounting evidence of its possible neuroprotective role.

To tackle this issue, Burdick and colleagues [87] recently evaluated the impact of lithium therapy on a large cohort of 262 subjects with BD, harnessing the power of a multisite, ongoing clinical trial of lithium monotherapy. The authors showed that while there were no differences at baseline between BD patients that were taking lithium compared with those that were not, a significant cognitive improvement in a number of neuropsychological tests was observed at follow-up in lithium-treated vs. lithium-free patients. The clinical relevance of this observation is enhanced by previous smaller studies that focused on a direct comparison between BD patients treated with lithium and those treated with anticonvulsants, given the widespread use of the latter to tackle BD symptoms. For example, Sabater and colleagues [88] showed in a study based on clinically stable BD patients that while lithium-treated patients presented a distinct neuropsychological profile, cognitive deficits were more widespread in subjects who were treated with anticonvulsants, either in monotherapy or if associated with other mood-stabilizing agents. Table 3 summarized the role of the most relevant pharmacologically available compounds on the link between BD and neurodegeneration.

While these observations seem promising for a paradigm shift in the conceptualization of a possible neuroprotective role for lithium, at least in a subset of BD patients, a key open issue remains to be assessed, i.e., the development of clinical, cognitive, or neuroimaging markers that could help to identify those BD patients who were more likely to benefit from the neuroprotective effects of chronic lithium therapy.

## 4. Neural Circuits of BD as Revealed by Neuroimaging Techniques: A Comprehensive Perspective

According to the most relevant study findings, the present critical review supported the existence of neuroprogression in BD. Importantly, BD populations with unfavorable clinical outcomes were documented to have poorer cognitive performance, indicating that neurological correlates are robustly associated with illness progression [89,90]. Molecular evidence derived from neuroimaging studies confirmed the neuroprogressive hypothesis of BD as well [56,57]. Overall, both clinical and biological factors strongly indicated the neuroprogressive nature of BD, with the actual models indicating abnormalities of the fronto–limbic neural circuits (e.g., the hippocampus and insula), their connections to anxiety and fear circuitry (i.e., amygdala), and their interaction with cognitive control regions (e.g., inferior frontal gyrus and anterior cingulate cortex) as key relevant brain regions as being implicated in the emotional and cognitive dysregulation in BD [91,92].

Based on our findings, neuroimaging studies provide the most consistent evidence supporting the neuroprogressive hypothesis in BD. Traditionally, both structural and functional MRI studies have increasingly supported, respectively, the findings of morphological abnormalities in fronto–limbic and subcortical structures and the overactivation in the amygdala and other limbic structures with functional and morphological differences in functionally related regions in BD [93,94,95].

In addition, a variety of studies underscored the presence of specific neurobiological alterations in relation to prior illness, the duration, and the number of episodes. In particular, volumetric studies demonstrated deficits in the volume of the prefrontal cortex, ventral prefrontal cortex, and anterior cingulate with a greater number of prior episodes. Similarly, a decrease in the subgenual prefrontal cortex, thalamus, and hippocampus volumes; disruption of cellular integrity in prefrontal or striatal areas; increases in lateral ventricle volume; increases in hyperintense lesions were also observed [96,97]. Moreover, in a 6-year longitudinal assessment, Abé et al. [98] underscored decreased frontal cortical volume (dorsolateral prefrontal and inferior frontal cortex), based on MRI findings in the mania group of BD patients compared to those without mania, suggesting that the reduced volume in brain frontal regions may be attributed to manic episodes.

Existing reports supported the altered structure–function relations, which may even significantly differ between bipolar subtypes [99]. In particular, BD type II showed a key relevant correlation between executive dysfunctions and reduced T2-weighted MRI cortical thickness in the right medial superior frontal cortex, left and right inferior precentral cortex extending into the right caudal middle frontal cortex, and medial occipital regions. By contrast, patients with BD type I had a less pronounced association, suggesting cortical dysfunction-related compensation mechanisms, and subtype-related neurobiological and cognitive profiles that warrant further investigation. It is noteworthy that smaller hippocampal volumes and decreased temporal lobe grey matter were reported in BD patients when compared to subjects with better clinical courses [100]. Recently, MRI data confirmed post-mortem findings of smaller hippocampal volumes in patients with BD compared to HC, especially in the cornu ammonis (CA) three-dentate gyrus subregion, and the subicular complex [101,102]. In addition, Han et al. [103] showed hippocampal subfield volume reductions in both major depressive disorder (MDD) and BD, a finding that was more prominent in MDD. Notably, significantly reduced volumes in the right CA, the granule cell layer (GCL), and the whole hippocampus were observed in BD patients, while the inverse correlation between BD illness duration and hippocampal subfield volume was reported as further evidence of the neuroprogressive nature of BD. Furthermore, the amygdala traditionally played a central role in models of emotion regulation in BD disorders, and increased amygdala volume was consistent with the direction of endocrine evidence of chronic cortisol hypersecretion in BD patients [104].

It is documented that emotional stimuli are also dependent on top-down neural systems, such as the prefrontal cortex, which control executive functions [105,106]. In line with this notion, functional underactivation in the dorsolateral, ventrolateral, ventromedial, inferior frontal, and subgenual prefrontal cortex (PFC) during both emotional and cognitive control have indeed been documented in BD [91,107], along with underactivation of the anterior cingulated cortex during cognitive control tasks in euthymic patients [108]. Similarly, it was hypothesized that cortisol hypersecretion in BD patients is consistent with the direction of the volumetric increase of the amygdala and inhibitory effects on the hippocampus [109].

Moreover, there is substantial evidence showing that early white matter changes, as well as trajectory-related gray matter alterations, including prefrontal, parietal, temporal cortex, and limbic structures, seem to be altered over the course of BD, which are especially associated with the number of episodes and length of the disease [110]. These findings support the notion that the damage of white matter tracts connecting cortical areas might hamper the connectivity between mood and cognition. Indeed, Besga et al. [111] previously identified the inferior longitudinal fasciculus that connects the occipital and temporal lobe structures, including the amygdala, hippocampus, and parahippocampus as the discriminant tracts compromised in both Alzheimer’s disease and BD, supporting how connectivity disruptions within the association fiber tracts could mediate both mood dysregulation and cognitive disturbances in these patients. To date, scant data has indicated a decreased volume in the cerebellum and vermis of BD patients, observed as a function of the number of prior episodes. Similarly, a further correlation with increased glucose metabolism, as measured using positron emission tomography (PET) in BD patients with treatment-refractory regimes was also reported [112]. Moreover, a paucity of data recognized the presence of oligodendroglial dysfunction and mitochondrial dysfunction embedded in white matter tract alterations [84] as neurobiological correlates in BD.

Recent neuroimaging studies using modern techniques, such as optical coherence tomography (OCT) imaging of the retina in BD patients, revealed neurogenerative changes in the central nervous system and, in particular, as an extension of the brain, underscored that the thinning in the peripapillary retinal nerve fiber layer (RNFL) and ganglion cell complex (GCC) layers of the peripapillary areas may reflect abnormalities of the limbic–thalamocortical pathways and glutamatergic dysfunctions in the thalamus and hippocampus with retrograde axonal degeneration in the retina of patients with BD [113].

In order to draw a more comprehensive conceptual framework, Perry and Miller [114] suggested that BD and its neuroprogression may be shifted toward a connectivity-based approach, emphasizing the disconnection syndromes as the paradigm for positioning BD disorder and dementia. Specifically, recent studies [115] also reported a smaller theta band connectivity strength modulation which was common to schizophrenia and bipolar patients and likely unrelated to pharmacological treatment.

Indeed, classic theories of brain functions in BD focused on separated and specialized anatomo-functional brain regions. Specifically, the role of large-scale circuits and networks has been described more recently [116]. In summary, the affected subnetworks in BD suggest a hierarchical network dysfunction, including the prefrontal and limbic areas, the anterior and posterior insula with its projections, the orbitofrontal cortex responsible for both cognitive and emotional dysregulation, and the fear circuitry of amygdala. Table 4 reported additional neurological and imaging correlates supporting the neuroprogressive hypothesis of BD.

The present review needs to be considered in light of the following shortcomings. First, it was not possible to perform a meta-analysis given the paucity of data related to the main topic. Moreover, studies recruited samples that referred to the different degrees of neuroprogression in BD, different outcome measures, and different assessments at various time points of the illness.

Furthermore, the inclusion and exclusion of studies within the present paper may, at least partially, reflect the authors’ choice, according to their expertise, despite this review being aimed at selectively summarizing the most important research in this specific field. In addition, some of the included studies suffer from small sample sizes and the longitudinal investigation of cases that develop chronicity and negative illness trajectory over time is not reported in all studies. Importantly, a number of methodological caveats (e.g., the different length of specific follow-up periods) resulted in findings that may be difficult to interpret. Furthermore, some of the included studies used retrospective designs or had a lack of strategies to ensure both inter-rater reliability and data validity. Moreover, excluding ambiguous BD cases of death (e.g., died by suicide) may result in an underestimation of the neuroprogression hypothesis underlying BD. Additionally, most of the investigated studies referred to a mixture of age groups; further research should take the age of BD patients into account when considering the validity of the neuroprogression hypothesis in BD. Another relevant limitation may be represented by the existence of heterogeneous samples and the inclusion of subjects at different stages of their illness. Specifically, there are, to the best of our knowledge, no studies in the current literature on brain functioning in BD that have taken into account the participants’ prior level of functioning.

Moreover, some studies focused on BD outpatients, who are usually more likely to manifest a less severe psychiatric condition relative to other patient populations and they did not collect (or only partially explored) the issue of neuroprogression in BD inpatients, focusing only on a few individual variables or without considering a specific control group. Finally, although various antidepressant medications demonstrated their efficacy in acute major depression [117,118] and are also able to enhance neuroplasticity mechanisms and adult neurogenesis in the hippocampus and prefrontal cortex [117], their use and differential effects in terms of neuroprogression remain controversial and poorly understood.

In summary, based on the most relevant study findings, this critical overview sustains the hypothesis regarding the existence of neuroprogression in BD. In particular, most of the existing neurocognitive, neuroimaging, and molecular evidence about the main topic demonstrated the existence of a neuroprogressive course of BD, at least in a specific subgroup of patients. Future studies using more reliable and targeted techniques are needed in order to replicate the present results and further support the direct link between neuroprogression and BD, chronicity, and disability.

## Figures and Tables

**Table 1:** Most relevant neurocognitive correlates of neurodegeneration in BD according to the current literature.

Study	Study Design	Sample Size	Limitations	Conclusions
[25]	Cross-sectional	30 euthymic type I BD patients; 30 HC	(i) Small sample size. (ii) 18/30 patients were on lithium medication, which could adversely affect cognitive performance.	Impairment of sustained attention may be considered as a neuropsychological marker for BD.
[26]	Cross-sectional	25 euthymic, remitted type I BD patients; 25 HC	(i) Small sample size. (ii) 20/25 patients were on lithium medication. (iii) The effect of mood stabilizers on encoding strategies was not investigated.	The number of depressive and/or manic episodes, but not illness duration itself, was associated with impairments in organization abilities and non-verbal memory.
[24]	Cross-sectional	40 euthymic BD patients; 30 HC	(i) Small sample size. (ii) Attention assessment was missing	A worse prior course of illness was related to poorer performance in delayed verbal memory and executive functions.
[28]	Cross-sectional	45 newly diagnosed type I BD patients; 25 HC	(i) Both BD patients and HC had average or higher than average premorbid/current intellectual functioning. (ii) No correlation between cognitive performance and time elapsed between ratings and cognitive testing was found.	The neuropsychological deficit was present in clinically stable BD patients.
[43]	Longitudinal	70 euthymic type I BD patients (49 with and 21 without significant cognitive impairment)	(i) All patients were taking psychotropic medications. (ii) Differences in impaired cognitive domains may not be equivalent in terms of the risk of recurrence.	Cognitive impairment might be considered the cause rather than the consequence of the clinical course.
[38]	Longitudinal	57 BD patients; 31 HC	(i) Small sample size. (ii) Confounding effects of substance abuse/dependence.	Patients who were euthymic for one year after the illness onset had better cognitive performance compared to subjects with a recurrence of mood alterations.
[53]	Cross-sectional	55 type I-II BD patients; 31 HC	(i) Small sample size of the subgroups. (ii) Influence of psychotropic medications. (iii) Exclusion of mixed episodes as polarity specifiers.	Manic polarity was associated with a greater cognitive impairment compared to BD with prevalent depressive polarity, regardless of the psychotic symptoms.

Note: bipolar disorder (BD), healthy controls (HC).

**Table 2:** The most relevant neuroimaging evidence concerning neuroprogression in BD.

Study	Study Design	Sample Size	Technique	Limitations	Conclusions
[58]	Cross-sectional	19 affective BD patients	CT	(i) HC sample was missing. (ii) Small sample size. (iii) Results only in subjects of 50 years of age or older.	A significant association between normalized ventricular volume and age was found.
[70]	Cross-sectional	11 BD patients; 15 HC	2.0 Tesla MRI	(i) Small sample size.	Reductions in fronto–temporo–limbic network regions were associated with poor clinical outcomes.
[65]	Longitudinal	9 BD children; 8 HC	1.5 Tesla MRI	(i) Small sample size. (ii) 6/9 patients were on lithium/mood stabilizers; (iii) All the children at the initial screening had psychotic features.	Increase in grey matter volume in the ventrolateral prefrontal cortex and temporal lobe structures.
[68]	Longitudinal	20 type I BD patients; 21 HC	1.5 Tesla MRI	(i) Small sample size. (ii) None of the participants were medically untreated.	The relative stability of the amygdala volume and hippocampal abnormalities was associated with poor cognitive outcomes over time in adults with BD.
[63]	Longitudinal	10 type I BD patients; 8 HC	1.5 Tesla MRI	(i) Small sample size. (ii) Wide age range (10–21 years old). (iii) Medication exposure. (iv) Variable interscan interval between groups.	The progressive prefrontal cortex volume loss was associated with disease severity in adolescents over two years.
[66]	Longitudinal	58 BD patients; 48 HC	4.0 Tesla MRI	(i) 48/58 patients were medically treated. (ii) Lack of assessment of the effect of illness course and duration, as well as medication status on structural changes.	The gray matter volume was increased in portions of the ventrolateral prefrontal cortex and hippocampus complex, centering on the parahippocampal gyrus but extending into the amygdala in BD patients.
[64]	Longitudinal	16 BD patients; 70 HC	1.5 Tesla MRI	(i) Small sample size. (ii) Analysis restricted to brain lobes. (iii) Patients had psychotic symptoms.	Progressive prefrontal cortex volume loss is associated with disease severity as assessed with the frequency of hospitalizations.
[71]	Cross-sectional	125 type I BD patients; 305 HC	1.5 Tesla MRI	(i) Usage of only one scale to measure impulsivity. (ii) The DSM-IV criteria rather than the DSM 5 criteria were used to categorize the participants.	Orbito–frontal abnormalities were associated with impulsivity and higher suicidal behavior.

Note: bipolar disorder (BD); computerized tomography (CT); Diagnostic and Statistical Manual (DSM); healthy controls (HC); magnetic resonance imaging (MRI).

**Table 3:** BD and neurodegeneration: the role of pharmacological available compounds.

Study	Study Design	Sample Size	Technique	Treatment	Limitations	Conclusions
[74]	Longitudinal	Animal model	/	Li_2_CO_3_ (4 eq/kg/day), sodium VPA (400 mg/dg/day), or saline by twice-daily intraperitoneal injections for 9 days or 4 weeks.	/	Lithium seems to be able to increase levels of the neuroprotective protein *bcl-2* and promote neurite outgrowth.
[84]	Longitudinal	12 untreated BD patients; 9 HC	1.5 Tesla MRI	Four weeks of lithium administration (blinded) at therapeutic levels (0.8–l.2 meq/L).	(i) Small sample size.	The use of lithium was associated with an increase in a putative marker of neuronal integrity (N-acetyl-aspartate), both in HC and BD patients, providing in vivo evidence of its possible neurotropic effect.
[82,83]	Cross-sectional	11 untreated BD patients; 16 BD patients treated with lithium; 39 HC	1.5 Tesla MRI	Lithium monotherapy (for the lithium-treated group)	(i) Small sample size. (ii) Lack of a statistically significant correlation between cingulate volumes and specific clinical variables. (iii) The sample was composed of multiple-episode patients; thus, it is not clear whether the decreased anterior cingulate volume preceded the appearance of symptoms or appeared afterward.	Compared with untreated subjects, BD patients treated with lithium presented with increased grey matter volume in vivo, as seen using structural MRI-based whole-brain measures, and a decreased anterior cingulate volume.
[85]	Longitudinal	12 BD patients	1.5 Tesla MRI	Lithium	(i) Small sample size. (ii) Several subjects at the time of evaluation were treated with low dosages of other medications. (iii) The study did not contain a group of patients treated with medications other than lithium over the same period.	Progressively increased volume of the hippocampus after treatment with lithium, which was in association with improved verbal memory.
[87]	Cross-sectional + longitudinal	262 BD patients (93 untreated, 169 lithium monotherapy)	/	Lithium	(i) Lack of a placebo control. (ii) Brief neurocognitive battery. (iii) Lack of cognitive testing available at a second time point in those patients who did not clinically respond to lithium.	Results from both cross-sectional and longitudinal analyses showed that lithium treatment did not significantly impair neurocognitive functions in patients with BD.

Note: B-cell lymphoma/leukemia-2 gene (*bcl-2*); bipolar disorder (BD); healthy controls (HC); valproic acid (VPA).

**Table 4:** Further neurological and imaging correlates supporting the neuroprogressive hypothesis of BD.

Study	Study Design	Sample Size	Technique	Limitations	Conclusions
[111]	Cross-sectional	12 BD patients; 25 HC	1.5 Tesla MRI; DTI	(i) Small sample size.	Fronto–striatal and temporal abnormalities have been observed in subjects with BD.
[107]	Cross-sectional	32 euthymic type I BD patients; 30 HC	fMRI	(i) One-third ratio of unmedicated patients. (ii) Use of a block design, in which blocks of go-only events were contrasted against blocks composed of both go and no-go events presented randomly. (iii) The relationship between regional changes in basal ganglia and IFC could not be determined. (iv) The vulnerability for future episodes and activation of the IFC and striatal regions remain to be explored.	IFC, cingulate, and striatal structures were activated in BD patients while performing a response inhibition task.
[102]	Cross-sectional	188 psychotic BD patients; 337 HC	MRI	(i) Confounding effect of medications. (ii) Imaging parameters were not optimized for imaging hippocampal subfields.	Widespread volumetric reductions in the hippocampus and its subfields within the spectrum of psychosis.
[101]	Cross-sectional	117 type I BD patients, 66 type II BD patients; 300 HC	1.5 Tesla MRI	(i) Possible confounding effects due to medication. (ii) The Bonferroni correction may be overly conservative because the hippocampal subfield volumes are not independent, and type II errors might have occurred. (iii) Using 1.5T MRI might have decreased the sensitivity to disease-related biological variability.	Smaller in vivo volumes of the hippocampal subfields CA2/3, CA4/DG, subiculum, and right CA1, along with smaller subiculum volume related to poorer immediate and delayed verbal recall in BD patients.
[98]	Longitudinal	31 type I BD patients	1.5 Tesla MRI	(i) Small sample size. (ii) Whole-brain analyses with a higher regional resolution, including the investigation of subcortical volumes, whereas larger study groups would give more detailed information on the effects of manic episodes on structural brain changes. iii) Lack of control data. iv) Probable presence of confounding factors given by differences in diet and exercise and undocumented drug use, as well as genetic, social, and environmental factors.	Progressive frontal cortical abnormalities were strongly related to manic episodes. Thirteen BD patients, labeled as the mania group, showed a decrease in cortical volume and area in both the dorso–lateral prefrontal cortex and inferior frontal cortex.

Note: bipolar disorder (BD); diffusion tensor imaging (DTI); functional magnetic resonance imaging (fMRI); healthy controls (HC); Inferior frontal cortex (IFC); magnetic resonance imaging (MRI).
